# Moroccan used cars dataset: Insights into the used car market

**DOI:** 10.1016/j.dib.2025.112087

**Published:** 2025-09-19

**Authors:** Essaadia Tabarnoust, Mohammed Mghari, Youssef Zaz

**Affiliations:** Abdelmalek Essaadi University, Faculty of Science, Computer Science Department, Tetuan, Morocco

**Keywords:** Used cars, Machine learning, Price prediction, Pricing trends, Vehicle attributes, Consumer behaviour, Data analysis, Moroccan market dynamics

## Abstract

The Moroccan used car market is a key indicator of consumer preferences, economic trends, and market dynamics in North Africa. This paper introduces the Moroccan Used Cars Dataset (MUCars-2024), which documents the used car market in Morocco throughout 2024. The dataset offers a detailed view of vehicles listed for sale during this period, providing valuable insights into this rapidly evolving market. Data were collected from prominent online platforms dedicated to car sales using web scraping techniques, ensuring comprehensive coverage. Post-collection, data were rigorously preprocessed, which included removing unreliable features with excessive missing data and standardizing other attributes such as price, mileage, and vehicle characteristics. The final dataset contains 101,896 listings, offering a robust representation of the Moroccan used car market.

MUCars-2024 provides a rich set of features—including vehicle specifications (brand, model, year), condition (mileage, first-owner status), and technical details (fiscal power, gearbox)—that enable detailed analysis. As a versatile resource for disciplines like economics, artificial intelligence, and automotive studies, it allows researchers to develop price prediction models, perform clustering analyses, and conduct spatial studies on consumer demand.

The MUCars-2024 dataset provides a high-resolution snapshot of a critical year in Morocco's automotive market. It serves as a foundational baseline for future temporal studies and enables immediate cross-market comparisons. As a publicly accessible resource, it directly supports research reproducibility and fosters innovation by bridging the gap between raw market data and academic inquiry, offering a valuable tool for data-driven research and industry practice.

Specifications Table

**Table:** 

Subject	Computer science
Specific subject area	Moroccan used car market dataset (MUCars-2024) documenting vehicle listings, attributes, and trends for future analysis and research applications.
Type of data	Table, filtered, processed
Data collection	The data was collected from online platforms selling used cars in Morocco using web scraping with Selenium, a popular Python tool for automating web browsing. The scraper extracted relevant car attributes such as model, price, year, mileage, and more. No specific inclusion or exclusion criteria were applied beyond filtering out incomplete listings.
Data source location	The dataset is located at: • Institution: Abdelmalek Essaadi University • City/Town/Region: Tetuan • Country: Morocco
Data accessibility	Repository name: Mendeley Data Data identification number: 10.17632/vjrbcb2rrt.2 Direct URL to data: Moroccan Used Cars Dataset (MUCars-2024) The dataset is publicly available on Mendeley Data under a Creative Commons Attribution 4.0 International (CC-BY 4.0) license, which permits reuse with appropriate citation.
Related research article	None.

## 1 Value of the Data

• **Dataset Overview**
  The dataset consists of over 101,896 listings, featuring essential vehicle attributes such as model, price, year, mileage, and condition. This comprehensive data enables an analysis of pricing trends, consumer behavior, and the impact of various car features on market demand.
• **Supports Predictive and Analytical Research**
  This dataset serves as a resource for developing machine learning models aimed at price prediction, market segmentation, and demand forecasting. Researchers can explore the relationships between car attributes and pricing dynamics to gain deeper insights into the market.
• **Practical Value for Industry Professionals**
  Automotive professionals, including dealers and analysts, can utilize the dataset to guide pricing strategies, inventory management, and regional market analysis. By understanding consumer preferences and market trends, professionals can tailor their approach to meet demand more effectively.
• **Geographic and Regional Analysis**
• This dataset enables geographic analysis of car pricing and demand patterns, by incorporating location-based data, offering valuable insights into regional market differences and local market dynamics across Morocco

## 2 Background

The used car market in Morocco plays a crucial role in the economy, providing affordable transportation amidst the high cost of new vehicles. Due to tax policies and an aging fleet, many transactions take place in the second-hand market, which also contributes to job creation in areas like sales, maintenance, and financing [1]. Analyzing this market offers insights into addressing environmental concerns, such as increased emissions, while supporting sustainability by extending vehicle lifecycles [2].

[1,2] The application of data mining for analysis and prediction is a well-established field, with comprehensive reviews detailing various techniques [3]. Recent studies have employed machine learning for price prediction in this sector. For instance, a study [2] using 18,000 records achieved a 90.7 % R² score with XGBoost, focusing on variables such as age, mileage, and region. The advancement of large language models like GPT-4 has also broadened the horizons for data processing and analysis in research [4]. However, datasets in these studies have limitations in scope and feature coverage. The 2024 dataset presented here expands upon this by incorporating additional features, such as model, fiscal power, gearbox type, and sector, and covers 101,896 records sourced from multiple platforms. Unlike previous datasets, this dataset is publicly available on Mendeley Data. This data offers broader opportunities for research on market trends, price prediction, and geographic variation in vehicle demand.

## 3 Data Description

The MUCars-2024 dataset is represented as a single CSV file and contains detailed information about used cars in Morocco. The dataset includes the following fields, each representing an attribute of the cars listed:

**Table:** 

Field name	Description
Brand	Manufacturer of the car (e.g., Dacia, Volkswagen)
Model	Specific version of the car (e.g., Polo, Sandero)
Year	Year in which the car was manufactured
Condition	Car's current state (e.g., excellent, good)
Mileage	Total distance the car has travelled, in kilometres
Gearbox	Type of transmission system (e.g., manual, automatic)
Fiscal Power	Engine capacity
Fuel	Type of fuel the car uses (e.g., diesel, electric)
Equipment	Features and options available in the car (e.g., GPS, airbags)
Number of doors	Number of doors of the car
Origin	Origin of the car (e.g., local, imported)
First owner	Whether the car is being sold by its first owner
Location	Geographic area where the car is located
Sector	Region where the car has been used
Price	Selling price of the car

• Dataset File Overview

○ File Format: CSV (Comma-Separated Values)

○ File Name: cars_dataframe.csv

○ Fields: 15 columns, as described above.

○ Number of Records: 101,896

• Dataset statistics

○ Missing values distribution

Visual representation of missing values in the final dataset. This heatmap displays the data sparsity across all 101,896 records. It was generated after columns with >40 % missing data were removed, illustrating the structure of the remaining missing entries which were intentionally preserved for analysis

○ Most common year of car production

The diagram highlights the distribution of cars in the Moroccan used cars market by their year of production, based on our dataset of used cars available for sale in 2024.

○ Top 10 brands distribution

The bar chart displays the top 10 most popular car manufacturers in the Moroccan used car market, reflecting brand dominance in 2024 listings.

○ Origin of Car Distribution

This distribution offers meaningful insights into the proportion of local versus imported vehicles. Notably, the ‘WW in Morocco’ category refers to vehicles with a temporary registration assigned to newly purchased or imported cars that have not yet completed the full registration process. Understanding this sheds light on the accessibility of these cars to potential buyers based on their clearance status and readiness for use in the local market

○ Condition distribution

This chart illustrates the distribution of cars based on their condition (good, excellent, or damaged…) It provides insights into the condition trends of vehicles listed for sale in 2024, offering a clearer view of the range of options available to buyers.

## 4 Experimental Design, Materials and Methods

The MUCars-2024 dataset was compiled to support research on Moroccan license plate recognition and vehicle market trends. Web scraping methods were employed to gather structured data on various car attributes from popular online platforms in Morocco. Extracting relevant information from the raw data required significant time and effort and involved several key steps:

• Data Collection and processing:

1. **Choosing Sources**: Reliable online platforms selling used cars in Morocco were selected for data collection.
2. **Web Scraping**: Python libraries were used for automating data extraction. The tools and techniques employed included:
  a. **Selenium**: Used to load web pages and interact with them, like clicking buttons or selecting filters.
  b. **Pandas**: Used to organize the data into a CSV file, making it easier to work with.

3. **Scraping Process**:
  a. **Loading Pages**: Selenium facilitated interaction with web pages, applying filters for car attributes (e.g., brand, price, and location).
  b. **Extracting Data**: Key information (e.g., car model, price, mileage, fuel type) was extracted from each car listing.
  c. **Handling Multiple Pages**: The scraper was designed to paginate through listings to gather complete data.
  d. **Saving Data**: The data was saved in CSV format for further use.

4. **Data Pre-processing**:
  a. **Handling Missing Values**: The primary step in managing missing data was to evaluate the completeness of each feature. Any column containing >40 % missing values was removed from the dataset, as it was deemed too sparse to be reliable for analysis. No further imputation or deletion was performed on the remaining missing values in the dataset. We intentionally preserved these gaps to provide a true representation of the raw scraped data, allowing researchers to apply their own specific handling techniques. This process resulted in the final dataset of 101,896 records.
  b. **Cleaning the Data**: To enforce consistent formatting post-scraping, we applied several pre-processing steps. Regular expressions (regex) were used to extract and standardize numeric fields such as Price and Mileage by removing non-numeric characters and converting strings to integers. For categorical fields such as Condition and Gearbox, we defined and applied a mapping dictionary to normalize variations in user input (e.g., “gd”, “Good”, “good” → “Good”). After automated standardization, a manual review of a random sample was conducted to verify formatting consistency and detect outliers. These steps improved metadata quality and ensured uniformity across key attributes.
  c. **Brand name normalization using GPT**: To ensure consistency in car brand names within the dataset, we addressed a high level of user-generated variation, including phonetic misspellings (e.g., mircidis, siat, bintli) and Arabic or Darija inputs (e.g., ). Such inconsistencies hindered proper grouping and analysis of vehicles by brand. We extracted all unique brand name entries from the dataset and used OpenAI’s ChatGPT (GPT-4) to generate standardized names. Each entry was provided to the model with the following prompt: *“Correct and standardize this car brand name for use in a Moroccan automotive dataset. Account for misspellings and Arabic transliterations: 〈****raw brand〉***.

**Table:** 

This approach allowed us to retain semantically accurate brand names while ensuring uniformity across all records, thus improving the quality of any brand-based analysis.

d. **Translation and Location Corrections**: Information was translated to English where necessary, and place names were standardized to align with standard naming conventions, improving the dataset's reliability for further analysis.
e. **Final Dataset**: The final dataset, stored in a csv file, contains the following fields: brand, model, condition, mileage, gearbox, fiscal power, fuel, equipment, number of doors, origin, first owner, location, sector, and price.

## Limitations

While the MUCars-2024 dataset provides a comprehensive snapshot of the Moroccan used car market, it has several limitations that users should consider.

• **Temporal Scope:** The dataset has a cross-sectional design, capturing market activity exclusively within the 2024 calendar year.
• **Potential Distribution Bias:** As the data originates from online platforms, the distribution of listings may be skewed towards urban centers and more popular vehicle models, reflecting the user base of these platforms.

## Future research directions

**Fig. 1:**
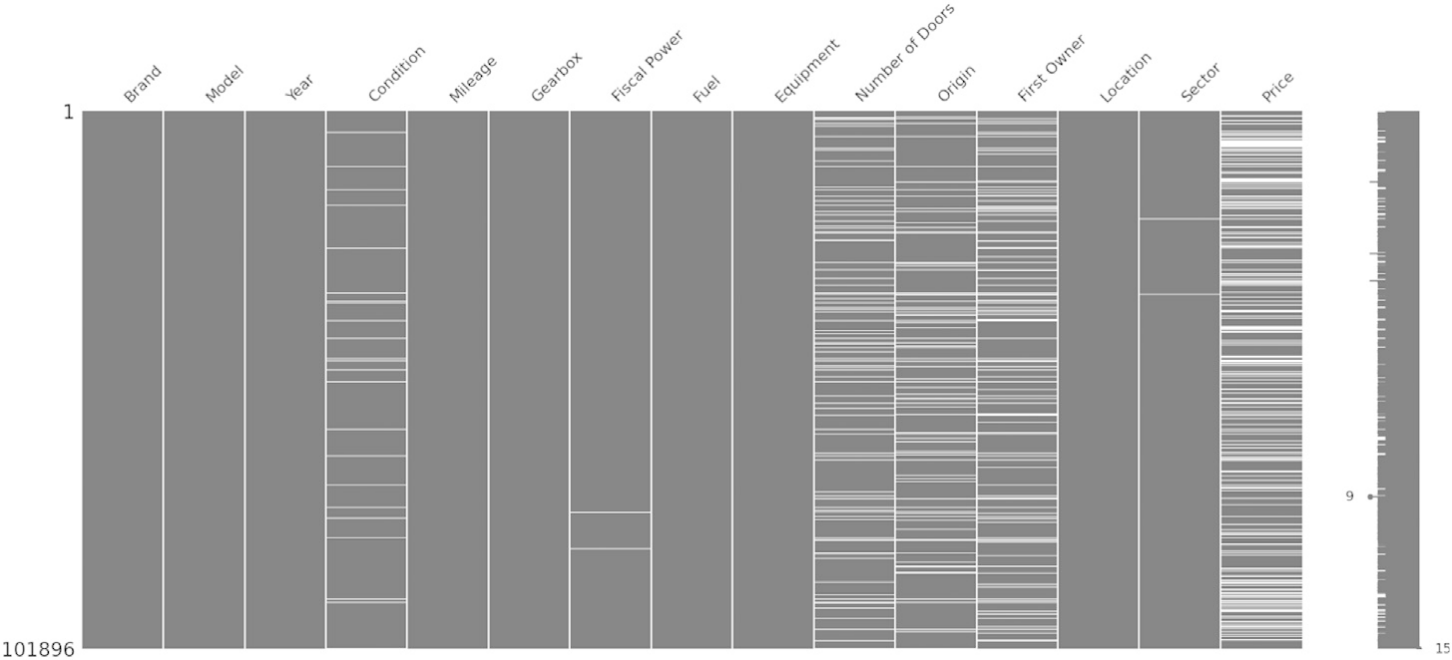
Missing values distribution.

**Fig. 2:**
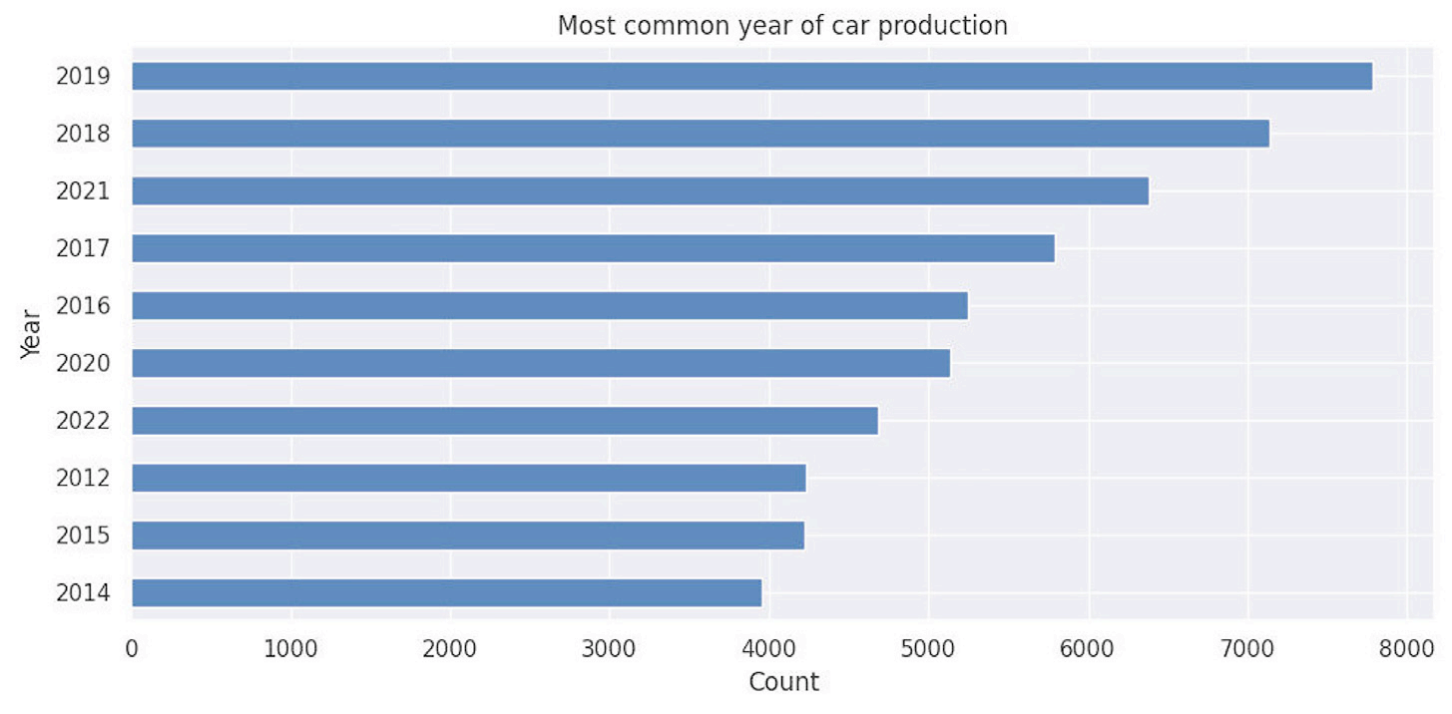
Most common year of car production.

**Fig. 3:**
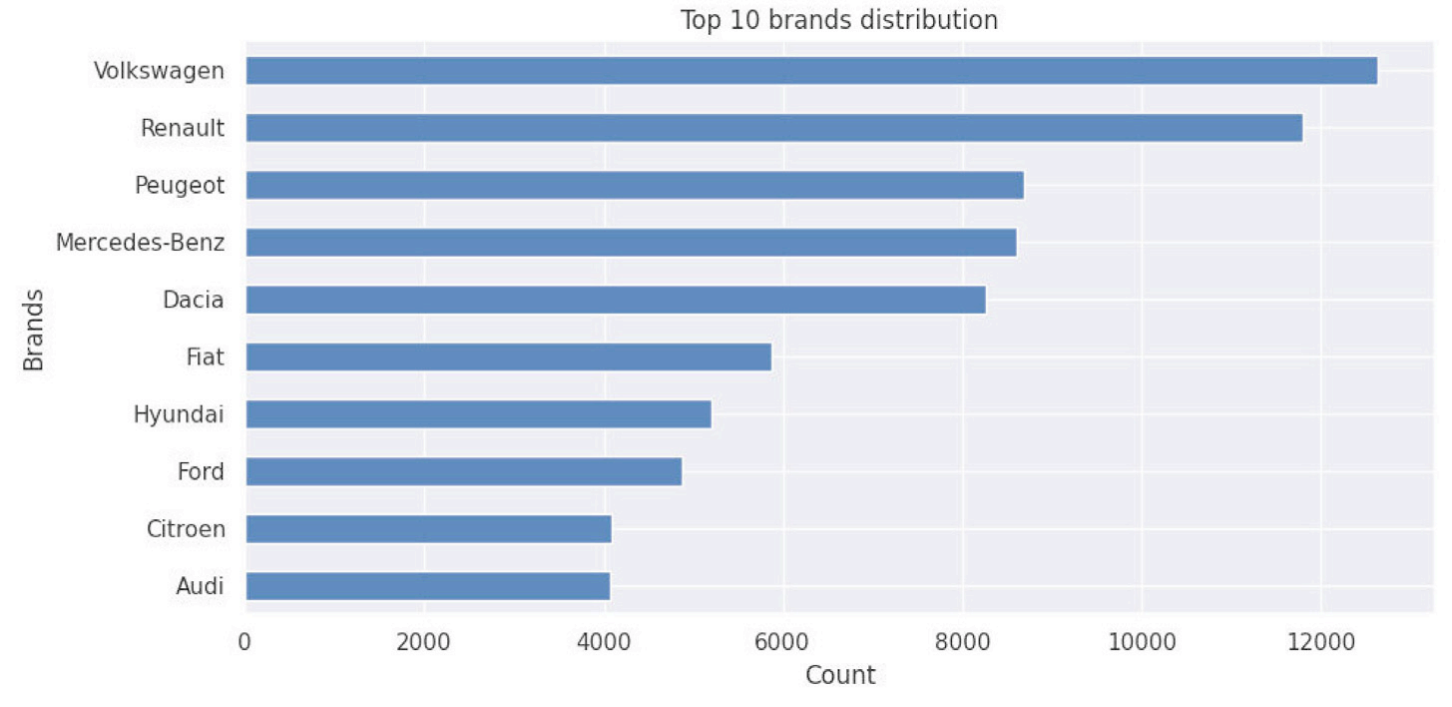
Top 10 brands distribution.

**Fig. 4:**
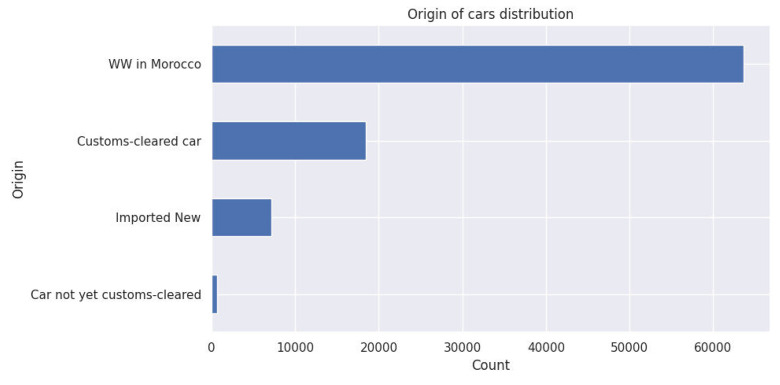
Origin of cars distribution.

**Fig. 5:**
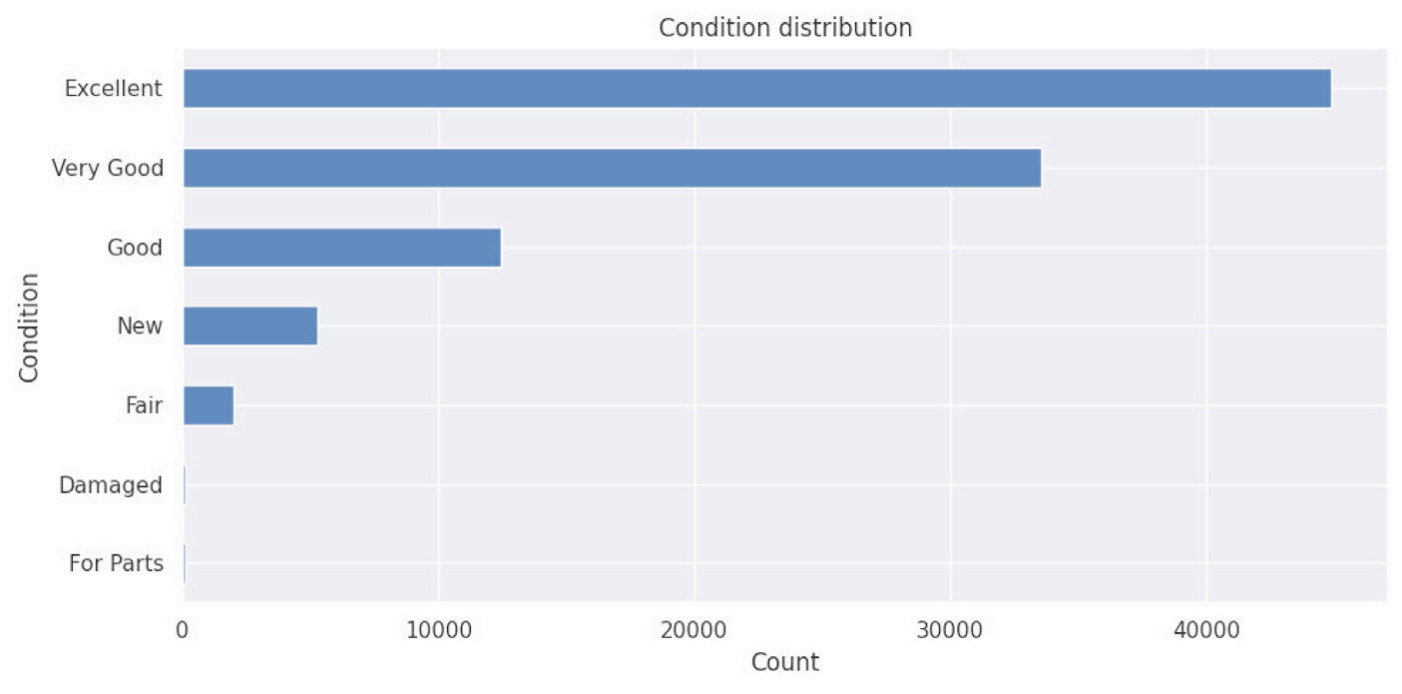
Condition distribution.

The MUCars-2024 dataset opens up several avenues for future research. A primary direction would be to conduct a longitudinal analysis by continuing data collection in subsequent years, allowing for the study of market dynamics and price evolution over time. Secondly, the dataset could be enriched by integrating external economic indicators, such as fuel prices, import tariffs, or inflation rates, to build more sophisticated predictive models. Finally, the ‘Equipment’ field, which contains unstructured text, is ripe for Natural Language Processing (NLP) techniques to extract specific features (e.g., “panoramic roof”, “leather seats”) and quantify their impact on vehicle pricing (Fig. 1, Fig. 2, Fig. 3, Fig. 4, Fig. 5).

## Ethics Statement

This dataset was collected using web scraping techniques, but no personal information, such as seller or owner names or contact details, is included. The data was anonymized, focusing solely on vehicle attributes, ensuring privacy and ethical compliance.

## CRediT Author Statement

**Essaadia Tabarnoust:** Writing – Original Draft, Investigation, Data Curation, Visualization, Writing – Review & Editing. **Mohammed Mghari:** Conceptualization, Methodology, Software, Data Curation, Writing – Review & Editing. **Youssef Zaz:** Supervision, Validation.

## Data Availability

MendeleyMoroccan Used Cars Dataset (MUCars 2024) (Original data).
